# Will Artificial Intelligence Translate Big Data Into Improved Medical Care or Be a Source of Confusing Intrusion? A Discussion Between a (Cautious) Physician Informatician and an (Optimistic) Medical Informatics Researcher

**DOI:** 10.2196/16272

**Published:** 2019-11-27

**Authors:** Qing Zeng-Treitler, Stuart J Nelson

**Affiliations:** 1 George Washington University Washington, DC, DC United States

**Keywords:** artificial intelligence, big data, data driven approach, medical informatics, digital health, digital medicine, quality of care

## Abstract

Artificial intelligence (AI), the computerized capability of doing tasks, which until recently was thought to be the exclusive domain of human intelligence, has demonstrated great strides in the past decade. The abilities to play games, provide piloting for an automobile, and respond to spoken language are remarkable successes. How are the challenges and opportunities of medicine different from these challenges and how can we best apply these data-driven techniques to patient care and outcomes? A New England Journal of Medicine paper published in 1980 suggested that more well-defined “specialized” tasks of medical care were more amenable to computer assistance, while the breadth of approach required for defining a problem and narrowing down the problem space was less so, and perhaps, unachievable. On the other hand, one can argue that the modern version of AI, which uses data-driven approaches, will be the most useful in tackling tasks such as outcome prediction that are often difficult for clinicians and patients. The ability today to collect large volumes of data about a single individual (eg, through a wearable device) and the accumulation of large datasets about multiple persons receiving medical care has the potential to apply to the care of individuals. As these techniques of analysis, enumeration, aggregation, and presentation are brought to bear in medicine, the question arises as to their utility and applicability in that domain. Early efforts in decision support were found to be helpful; as the systems proliferated, later experiences have shown difficulties such as alert fatigue and physician burnout becoming more prevalent. Will something similar arise from data-driven predictions? Will empowering patients by equipping them with information gained from data analysis help? Patients, providers, technology, and policymakers each have a role to play in the development and utilization of AI in medicine. Some of the challenges, opportunities, and tradeoffs implicit here are presented as a dialog between a clinician (SJN) and an informatician (QZT).

Drs Nelson and Zeng-Treitler work together at the Biomedical Informatics Center at George Washington University. In the following we present a hypothetical dialogue which grew out of discussions they had as they considered their differing viewpoints of how artificial intelligence (AI) has developed and where it is going. While Dr Zeng-Treitler’s view of the future of AI is highly optimistic, Dr Nelson's opinion is more cautious. Dr Nelson was a practicing academic internist who became involved in informatics many years ago. He collaborated with Scott Blois on RECONSIDER (an early clinical decision support system) and on the Unified Medical Language System (UMLS) project. He eventually moved to the National Library of Medicine as Head of Medical Subject Headings. While at the National Library of Medicine, he fathered RxNorm, while continuing his work on UMLS and projects involving UMLS. Dr Zeng-Treitler has a background in computer science and obtained her PhD in medical informatics from Columbia University. She has led a number of projects in clinical data mining, natural language processing, and consumer health informatics. During the past few years, her team has been actively investigating the use of AI techniques in clinical research including development of a novel explainable deep learning approach.

**Dr Zeng-Treitler (The "Optimist"):**

After decades of promises and disappointment, thanks to the seemingly unbounded computing resources and novel, data-driven methods, AI technology has finally arrived. From Jeopardy and Siri to face identification and autonomous vehicles, data-driven approaches have made the leap from laboratory experiments to applications that are transforming our lives outside health care. In some cases, these approaches have come close to passing the Turing Test—a test of a machine’s ability to exhibit supposed human-like intelligence; machines can now perform some complex tasks such as image recognition and authentic game play as well as or better than humans would. Some would argue that the requisite approach is decidedly nonhuman. However, whatever the means to achieving these innovations, such successes have not been followed by analogous successes in health care.

One dramatic example of this disparity of accomplishment is AlphaZero, a computer game engine that mastered chess, Shogi, and Go. Even before the arrival of the current generation of data-driven artifacts, chess engines have been shown to be able to play at a level superior to that of chess champions. Players of Go (a board game that is thought to be much more complex than chess), however, believed that computers were no match for high-level professionals in this game. This belief was shattered first by AlphaGo, which soundly defeated the reigning world champion of Go. Then came AlphaZero. The news is no longer that such approaches can beat the champions of Go, chess, or Shogi. Rather, the remarkable fact is that AlphaZero did not learn from human experiences and that it defeated the best prior chess engines such as Stockfish. AlphaZero triumphed by playing more games against itself than had ever been played by all human players. This is not an approach we could readily duplicate in health care.

**Dr Nelson (The "Cautious" One):**

Is winning a game, with defined rules and objectives, really the best test of human intelligence? In hindsight, the answer is “No.” For example, sophisticated chess playing programs have existed for nearly 50 years; from such programs, we learned how to organize computing resources to apply simple algorithms in a scalable way. Put differently, we did not learn anything about chess or how humans, even experts, play it. Instead, we learned that a supposed intelligence-requiring task was susceptible to a computational approach. We need to ask where and how such an approach is applicable in health care.

For example, when doing tasks that are generally thought to be human and creative, can the machine recognize when it is out of its depth? Sometimes, humans have the ability to do so. However, if we can define the realm closely enough, I agree that the machines can do wonders. So, how do we define the realm?

In Blois’ seminal paper on Clinical Judgment and Computers [1], he described the world of a physician’s thought process when seeing a patient, with the diagram shown in Figure 1. Point A for a physician would be where the patient walks in the door to be seen for the first time. The nature of the complaint, the context in which the complaint occurs, and all of the myriad possibilities are present. As the problem definition moves toward Point B, a computer is better able to manage the information and knowledge necessary for high-quality care. One way in which we can think about defining the realm is that we are moving toward Point B. Some computer scientists have argued that Point A is just about managing facts, but, as Blois observed, it is more about relevance—something that has proven difficult to replicate computationally.

**Figure 1:**
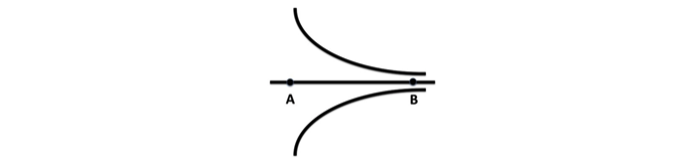
The cognitive span required during diagnosis.

**Dr Zeng-Treitler:**

It is indeed important to define the realm for an AI application. Many tasks in health care are much more complex than game play, and we have not witnessed the triumphs of analogous approaches in the biomedical domain as have been achieved in game play. Quite a few studies have been applying the latest deep learning technology (a key AI method) to biomedical datasets [2-4]. The specific applications included image processing, natural language processing, and risk prediction. Deep learning, compared with traditional statistical and machine learning methods, has often shown modest improvements rather than breakthroughs [5-7].

**Dr Nelson:**

Whatever the details of these approaches, they apply nearly unbounded computing resources to very large amounts of data, something that has yet to happen in health care. Therefore, these approaches might prove helpful, but we do not know for sure yet.

For example, a simple question posed by a colleague is beyond our current capabilities: Given a patient who starts out with a feature of metabolic syndrome, which feature of the syndrome will he or she tend to exhibit next? Simplistically, this is exactly the kind of challenge that a data-driven approach should help with, and yet, it is currently “over the horizon” due to the insufficient data that were collected in the past.

**Dr Zeng-Treitler:**

Data are a key challenge when applying data-driven approaches to patient care. To begin with, biomedical data are highly complex. There many different types of data including image, text, numerical values, categorical classifications, and DNA sequences, representing tens of thousands of lab tests, procedures, diagnoses, medications, genetic markers, etc. Each data type also has its own characteristics; for example, a laboratory test value may need to be interpreted in the context of age, gender, and current conditions. However, diagnostic codes for different diseases have varying levels of accuracies.

In biomedical data analysis, there is also the paradox of having too much and not enough data at the same time. On one hand, there are a tremendous amount of medical record, social media, and literature data. Efforts like the Million Veterans Project [8] have also collected a huge amount of DNA data. Using devices for tasks like activity tracking and continuous glucose monitoring generates more data than our current medical record systems can digest. On the other hand, the health record of a patient is an open system with much missing information in contrast with the closed system of a chess or Go game, where all data are available. Patients are observed at irregular intervals (eg, at clinic visits or during hospitalization) and are never subjected to all possible tests or treatments. Sometimes, death is the only definitive outcome.

**Dr Nelson:**

I agree that data types are multiple and complex. Simple solutions are insufficient, and the proliferation of irrelevant data in a record, not to mention the current cut-and-paste or fill-in-the-template fad, obscures what is important.

One of the major difficulties with medical data is not just that it is not enough, but also that it is theory laden, that is, very few pieces of data are recorded routinely. A lot of data in observations are gathered only when the clinician has thought it is appropriate, that is, when diseases are tested for their absence or presence. If there is no reason to do the test, the test is not performed. Only a few tests are ever performed routinely; a transcribed set of physical observations (as is done in physical examination) is rarely recorded in enough detail (not to mention the failure to observe, which often occurs) to provide sufficient data for a more comprehensive analysis. For that reason alone, studies based on the recorded observations are often incomplete and potentially misleading. However, for predictions, observations not made may be the critical ones. Think about the patient with metabolic syndrome mentioned above. What data are we missing?

Similarly, the results of clinical trials are not a complete picture. Even though participants have been selected, often excluding many individuals because of complicating conditions, the data collected on the participants are designed for testing certain hypotheses, with narrowly defined outcomes. A common criticism is that such trials are so artificial that they are irrelevant.

**Dr Zeng-Treitler:**

The lack of integrated and standardized datasets is another issue. Although we can find many large datasets, they are often incomplete and difficult to link to other information. For example, environmental exposure, diet, physical activity, and genetic profile are among the common missing pieces of information when we examine the records about an individual patient. Detailed clinical trial datasets tend to lack long-term follow-up. Privacy issues and monetary incentives are also obstacles in data integration efforts.

**Dr Nelson:**

Real semantic interoperability to integrate and standardize datasets requires support in both terminology and how that terminology is used. Currently, human intervention is often required to interpret what one system is saying for use in another system. This situation is unfortunate; we can hope that over time, the necessary connections will take place (think of how the United States went from operator assistance on every telephone call to the automatic switching that takes place today). Such a change can only happen when many people see the need for and implement a common standard.

In addition, the notion of “large” in the context of health care data is only relative. Think, instead, of many experiments undertaken by Google; if they desire, the amount of data that can be used to develop and test a model is orders of magnitude greater than that available in health care.

**Dr Zeng-Treitler:**

In the domains where data-driven approaches have demonstrated success, there are outcomes that can be judged by human experts or machines themselves. For example, bilingual speakers can tell if natural language translation is working well, and the outcome of board or computer games can be easily determined. This allows easier simulation or annotation of data for machine learning. Such a task is much harder in the biomedical domains; investigation of causes or treatments of diseases in humans involve costly and long-term studies. In some cases, ethical concerns prohibit the experiments; for example, the introduction of potentially harmful genetic mutations into healthy human subjects is out of the question. We lack long-term outcome data for many treatments.

**Dr Nelson:**

I am not sure that there ever will be such a gold standard without a completely arbitrary definition. Variation between individuals is also a major obstacle. Although we perform studies using multiple subjects to account for biologic variability, our results are only approximate in their relevance to a given individual. For example, assuring genetic diversity in clinical trials is challenging, to say the least. Even the simplest tasks can be staggeringly multifactorial; for example, the information content of genetic testing for warfarin metabolism can be outweighed by whether the patient had lettuce for lunch.

To expand on this observation, suppose you have an automobile that is not working appropriately. Today, you consult the sensors and the computer readout to give you very precise information about what is going wrong. The automobile has a specific design, with specific parameters that can be measured. All of the vehicles of the same year make and model can be assumed to be alike in those important aspects. It is important to realize that every human (with the exception of identical twins) is genetically unique. In that way, people are very different from automobiles or other mechanical devices. To compound the complexity with which the cause of a human problem can be addressed, what the individual experiences throughout their life is unique. Although we have nice abstractions or methods of identifying individuals who share some common characteristics (whether the presence or absence of a disease, the response or lack thereof to a medication, the similar environment, or other considerations), these are only a shorthand notation. With 7 billion persons currently living in this world, the problem appears almost open-ended. Too often in data analysis, we look at diagnostic codes as having a deep meaning. These are accepted without any recognition of the degree of uncertainty of the diagnosis. All our data may be helpful and useful, but we need to continue to view them with a large grain of salt. The fact that Google Translate works as well as it does gives us hope, but as complex as natural language translation is, it is simpler than some clinical tasks.

**Dr Zeng-Treitler:**

Despite these challenges, applying data-driven approaches has the potential to transform health care. Today’s health care is labor intensive, from scheduling and triage to diagnosis and treatment. Many tasks currently undertaken by humans can be carried out by intelligent software solutions supported by sufficient data. For instance, improved voice recognition and summarization technology might help reduce the amount of time patients and clinicians spend on paperwork. Improved decision support tools ought to be able to help patients decide about the appropriateness of seeking care. An accurate assessment of short- and long-term risks and benefits will inform treatment selection and lifestyle changes.

**Dr Nelson:**

To provide another use case, there is evidence that type II diabetes may be reversible, but it is hard to apply this knowledge to an individual patient. Given the patient in front of me, what should I do, or recommend, and with what expectation? Demographics, genomics, comorbidities, psyche, competing risks, and other medications, all play a role. How, in a given person, do I reconcile all the possibilities?

**Dr Zeng-Treitler:**

To develop these useful AI tools, we need better data, technology, and policy. To accumulate comprehensive, life-time data, patients must be in control and should be incentivized to share their data for research and care. Insurance, pharmaceutical, and medical institutions change over time. Currently, there are barriers for individuals to be the center of collecting the data on themselves. The barriers are present in data entry, collection, and storage; for example, some personalized health record products are tethered to an institution, while others require extensive transcribing efforts by patients or caregivers. Nevertheless, without patient consent and collaboration, gathering and linking longitudinal environmental, genetic, clinical, and behavioral data are neither feasible nor ethical. The current conditions are a huge barrier to any attempt to use data-driven approaches that have worked outside health care.

Efforts including PatienstLikeMe [9] and the All of Us Research Project of the National Institutes of Health [10] are examples of innovative approaches to curate bigger and better datasets. Most patients, however, are not engaged in such efforts. Patients are inherently motivated to improve their own health, but naturally have concerns regarding privacy and often do not see immediate benefits of participating in long-term studies. Appropriate incentives (eg, discounts for routine preventive care) coupled with security and authentication technologies are needed to entice a large and diverse population of patients to gather and share their data. The health care industry today owns parts of patient data and has limited motivation to purchase data from their customers. As the value of data increases, patients will become more valued as a partner.

**Dr Nelson:**

I agree that patients will need to assume the responsibility of carrying and sharing the information about themselves. However, experience tells us that not everyone is able or willing to do so. It will need cultural and political climate changes to encourage that development.

When we can collect data that are not directly what the philosophers would call “theory-laden,” we may be able to refine our crude methods of patient diagnosis and care. I look forward to that day. If patients are the carrier of those data, it will be easier to obtain and use for analysis.

**Dr Zeng-Treitler:**

We also need to design and implement methods specifically for handling very large and “messy” clinical data. For example, we need to understand the context of missing data and errors to get a better picture of ground truth. A lab result may be missed because there is no indication for it, practice preference, an alternative method of assessing, or a failure of data entry. Imagine how much more difficult a chess game will be, if a human player or a chess engine could only observe some squares on the board at irregular time intervals with some error or distortion of the observation.

Further, we do not have an operational definition of “ground truth” in health care; a simple proposal is that one feature of ground truth is that it has predictive value—something that will be valued by clinicians and patients alike.

**Dr Nelson:**

Google has demonstrated that they can use lots of data to predict likely values for missing data in other areas [11]; however, it is yet to be determined whether this might work in medicine, but it is probably worth a trial. Irrespective of whether we can use large volumes of data to impute missing values, exploring how to handle the problem of absent observations is crucial, especially whenever we try to apply the results of data-driven approaches to individual patients.

Another thought is that data that are missing, for any reason, are an observation in itself; the fact that the data were not obtained and recorded may be important. Think of the finding that the day and time of a test were more predictive of outcome than the result of the test [12]. We know that the data that are missing will have some predictive value.

**Dr Zeng-Treitler:**

On a different note, explanation of the data-driven models is critical to not only their adoption but also their impact [13]. Predicting that a patient will have certain adverse events in the next several days or years is desirable. It may be argued that it is even more important to know the modifiable factors that can reduce risk and enhance outcome. Since deep learning models can be highly nonlinear, we have the opportunity to discover novel and complex patterns.

**Dr Nelson:**

I agree that explaining the prediction is critical; it is something that separates health care from, say, recognizing whether an image is a dog or a cat. However, I think you meant to say predicting a patient will *probably* have some adverse outcome. Nothing in life is certain except that it will end. However, we can say “it appears this behavior or finding will likely have an effect on your future” and hopefully be able to express some degree of confidence in that prediction.

Learning how to express the confidence in a prediction is also important. How many folks really understand the statistics behind the predictions that occur today? What are the underlying assumptions behind any probabilistic model? It is more likely that with more frequent use and familiarity with the use of measures derived for AI models will lead to their acceptance.

**Dr Zeng-Treitler:**

I agree. These are all steps to be taken in order to optimize the use of big data through AI to improve medical care.

**Dr Nelson:**

As a parting thought, we need to be cautious about how intrusive data-driven approaches might be in the care process. Although McDonald [14] demonstrated that performance in care improves with reminders [14], the later experience has been one of too many reminders, leading to alert fatigue. When caregivers choose to override and ignore helpful information because of overload, have we accomplished anything?

I hope that careful design of systems and consideration of clinical workflow will alleviate the problem of excessive intrusiveness. Although it is tempting to just “let AI do it,” the recent experiences with the Boeing 737 MAX demonstrate that there is danger in doing so. Neither AI nor a pilot alone is the optimal strategy in flying. In health care, involving patients more extensively in their care, together with AI and providers, may ultimately be an approach that works.

## Abbreviations

AI: artificial intelligence
UMLS: Unified Medical Language System
